# Metabolic regulation of synaptic plasticity in anorexia nervosa

**DOI:** 10.3389/fnsyn.2026.1830809

**Published:** 2026-04-22

**Authors:** Olof Lagerlöf, Qiongxuan Lu, Manish Bhattacharjee, Rashmi Arora, Linkun Han, Jingyu Pan, Sabrina Galizia, Peter Asellus, Erik Ekbäck

**Affiliations:** 1 Department of Clinical Sciences, Umeå University, Umeå, Sweden; 2 Wallenberg Centre for Molecular Medicine, Umeå University, Umeå, Sweden; 3 Department of Medical Translational Biology, Umeå University, Umeå, Sweden; 4 Department of Public Health and Clinical Medicine, Umeå University, Umeå, Sweden

**Keywords:** anorexia nervosa, appetite regulation, food intake, metabolism, synapses, synaptic plasticity

## Abstract

Anorexia nervosa (AN) is increasingly understood as a metabo-psychiatric disorder in which metabolic biology and neural circuit function are intrinsically intertwined. Genetic studies reveal that AN is associated with heritable metabolic traits suggesting that metabolic vulnerability contributes to the disorder. The metabolic profile of AN further shapes brain responses; endocrine signals such as insulin, leptin, ghrelin, and adiponectin elicit atypical neural responses in circuits regulating appetite, reward, interoception, and cognitive control. This altered signaling is accompanied by circuit-specific remodeling, suggesting that the chronic metabolic dysregulation seen in AN affects synaptic plasticity across distributed brain regions. Neural systems that integrate metabolic, emotional, and cognitive information—including hypothalamic, striatal, prefrontal, and limbic circuits—show altered plasticity under starvation. Glucose and insulin modulate excitatory–inhibitory balance and synaptic efficacy, while ketone bodies act as starvation-associated neuromodulators influencing transmitter release and structural plasticity. These and other body-to-brain signals recalibrate network dynamics central to food intake, motivation, and learning. At the molecular level, intracellular metabolic sensors such as AMPK, mTOR, and O-GlcNAc function as transducers that convert nutrient availability into changes in protein synthesis, receptor trafficking, and dendritic spine architecture, providing mechanistic links between metabolic state and synaptic remodeling. Overall, converging evidence supports a model in which AN arises from interactions between metabolic traits and the plastic neural circuits mediating food intake, emotion, and cognition. By clarifying how metabolic signals reshape synaptic ensembles in AN, we present a framework for understanding mechanisms of vulnerability and identify targets capable of restoring adaptive plasticity. This review suggests a trajectory in which treatments jointly address metabolic physiology and brain-based processes of learning, motivation, and affect.

## 1 Introduction

Anorexia nervosa (AN) is an eating disorder in which people maintain dangerously low body weight through sustained restriction of food intake, intense fear of weight gain, and a distorted perception of their own body size and shape (Bulik et al., 2021, 2022). This leads to serious medical and psychosocial impairment and AN has one of the highest mortality rates among psychiatric disorders (Attia, 2010), with deaths resulting both from the physiological effects of starvation and from suicide. AN frequently follows a chronic, relapsing course beginning in adolescence or young adulthood (Bulik et al., 2021; Frank et al., 2019).

Until now, AN has been conceptualized primarily as a psychiatric disorder driven by sociocultural pressures to be thin, individual psychological factors such as perfectionism and anxiety, and maladaptive family and interpersonal dynamics. Metabolic dimensions of AN are often passively viewed as a consequence of starvation rather than a contributing cause (Bulik et al., 2022; Frank et al., 2019). Despite decades of psychotherapy-focused research, outcomes remain unsatisfactory for many patients. There are still no medications with clear, robust efficacy for core AN symptoms, suggesting that this purely psychological model is incomplete (Bulik et al., 2021; Mao et al., 2026).

Recent genetic, neuroimaging, and other data have begun to reveal the role of metabolic factors in AN, supporting the concept that it is as a “metabo-psychiatric” disorder. This term emphasizes that the illness spans both brain and body and that metabolic factors are deeply embedded in its etiology and maintenance rather than being only downstream consequences of weight loss (Bulik et al., 2021; Zhang and Dulawa, 2021).

In this review we will present an up-to-date presentation of the metabo-psychiatric nature of AN with converging observations from clinical studies and neuromolecular pathways identified in animal models. The evidence suggests that AN is driven at least in part by metabolic mechanisms that regulate how different parts of the brain communicate with each other through affecting synaptic plasticity.

## 2 Metabolic drivers of anorexia nervosa

Multiple lines of evidence indicate that metabolic factors can increase the risk of and maintain AN by affecting what and how different parts of the brain communicate with each other.

### 2.1 Genetic basis of metabolic disturbances in anorexia nervosa

Family and twin studies suggest that AN is more than 50 % genetically determined (Duncan et al., 2017; Watson et al., 2019). Early attempts to identify specific susceptibility genes had relatively small sample sizes and were limited in what targets they could identify. Recent genome-wide association studies (GWAS) have established AN as a polygenic disorder that is influenced by numerous common variants of small effect (Duncan et al., 2017; Watson et al., 2019). The first well-powered GWAS identified a genome-wide significant locus and showed broad genetic correlations with psychiatric phenotypes such as schizophrenia, traits like neuroticism, and with educational attainment, which indicates shared pathways across cognitive and psychiatric domains (Duncan et al., 2017). Importantly, strong negative genetic correlations were seen with body mass index, insulin, glucose, and lipid traits, along with positive correlations with high-density lipoprotein cholesterol, which demonstrated that AN-associated alleles align with a metabolically “lean” profile (Duncan et al., 2017).

A subsequent, larger GWAS including nearly 17,000 individuals with AN identified eight genome-wide significant loci and confirmed that the disorder’s genetic architecture bridges psychiatric and metabolic biology (Watson et al., 2019). This expanded previous results and demonstrated robust genetic correlations with obsessive-compulsive disorder, major depression and schizophrenia, while reproducing strong negative correlations with type 2 diabetes and body mass index and positive correlations with favorable lipid markers, even after controlling for anthropometric effects (Watson et al., 2019). These findings motivated the proposal that AN should be conceptualized as a “metabo-psychiatric” disorder rooted in both neurobiological and metabolic pathways (Watson et al., 2019).

More refined genetic analyses further support this conceptualization. Adams and Cairns (2026) showed that AN shares heritable components with metabolic traits even when statistically controlling for body mass index, indicating deeper biological relationships rather than mere reflections of thinness. Colocalization and regional genetic-correlation analyses identified genomic regions in which single variants influence both AN and metabolic phenotypes, highlighting pleiotropic biological pathways linking psychiatric and metabolic processes (Adams and Cairns, 2026). Makowski et al. (2025) extended this work to metabolomics and they found significant genetic correlations between AN and a wide panel of lipid-related metabolites, often in directions opposite to those seen with type 2 diabetes and body mass index, suggesting that AN-related alleles contribute to metabolomic profiles characteristic of lower adiposity and cardiometabolic protection.

Rare-variant analyses complement these polygenic findings by identifying specific metabolic genes that may exert large effects in subsets of patients. Lutter (2025) discovered a significantly increased burden of rare, predicted damaging variants in BBOX1—encoding the enzyme essential for carnitine biosynthesis and mitochondrial fatty-acid transport—among individuals with AN compared with population reference data. Because carnitine plays a critical role in energy metabolism, these findings suggest that impaired fatty-acid oxidation may contribute to disease susceptibility in some cases, reinforcing the metabolic dimension of AN’s genetic architecture.

Epigenetic evidence further supports interactions between genetic risk and metabolic state. Käver et al. (2024) showed that prolonged caloric restriction and stress—hallmark features of AN—may induce DNA methylation changes in genes regulating appetite, stress response and metabolic processes, indicating that starvation-driven endocrine and metabolic signals can shape gene-expression environments in ways that interact with inherited vulnerability.

Sex-specific metabolic genetics may also help explain AN’s female predominance. Hübel et al. (2019) showed that genetic variants associated with body-fat percentage and fat-free mass—traits with strong sex differences—exhibit sex-specific correlations with AN, with stronger associations in females. These findings suggest that sex-specific metabolic biology may partly contribute to the sex bias in incidence.

Taken together, converging evidence from common-variant GWAS, rare-variant sequencing, metabolomic genetics and epigenetic studies indicate that the genetic basis of AN is driven by an interplay between psychiatric susceptibility and metabolic biology (Duncan et al., 2017; Watson et al., 2019; Adams and Cairns, 2026; Makowski et al., 2025; Lutter, 2025; Käver et al., 2024; Hübel et al., 2019).

### 2.2 Anorexia nervosa affects how the brain responds to metabolic cues

AN is accompanied by systemic and neurobiological metabolic shifts that go beyond what would be expected from calorie restriction alone (Holsen et al., 2012). In addition to genetic studies, physiological research suggests that these metabolic alterations are not merely passive consequences of starvation but may actively drive core symptoms, including persistent food restriction despite low weight, altered hunger and satiety, and rigid, habit-like behavioral patterns (Rigaud et al., 2007; Simon et al., 2020; Wierenga et al., 2017).

Visual food-cue paradigms have been used to map the brain’s response to food. These food related cues reliably engage cognitive, emotional, and neural systems that are theoretically central to real-world eating behavior (Lloyd and Steinglass, 2018). Studies show that AN patients differ from healthy individuals in attention to, evaluation of, and emotional response to visual food stimuli. They reflect patterns of vigilance, avoidance, and altered hedonic appraisal that directly mirror the core clinical symptoms of food-related anxiety and restriction (Lloyd and Steinglass, 2018). For example, research by Bronleigh et al. (2022) show that patients exhibit atypical activation in salience, reward, and cognitive-control circuits—including the insula, anterior cingulate cortex, and prefrontal cortex—when viewing food stimuli, indicating that food engages a neural configuration distinct from that of healthy controls.

A complementary multimodal meta-analysis identified consistent reductions in gray-matter volume and resting-state activity in midline cingulate and occipito-parietal regions, areas involved in integrating visually salient and emotionally charged information (Su et al., 2021). It has also been shown that individuals with AN exhibit heightened activation in prefrontal, insular, amygdalar, and hypothalamic regions in response to food images, a pattern reflecting strong cognitive control and anxiety rather than reward-related engagement (Celeghin et al., 2023). This profile diverges from that of bulimia nervosa or binge-eating disorder, where food cues typically evoke reward-reactive responses in striatal regions, highlighting the disorder-specific nature of food-cue processing in AN (Celeghin et al., 2023).

**Figure 1:**
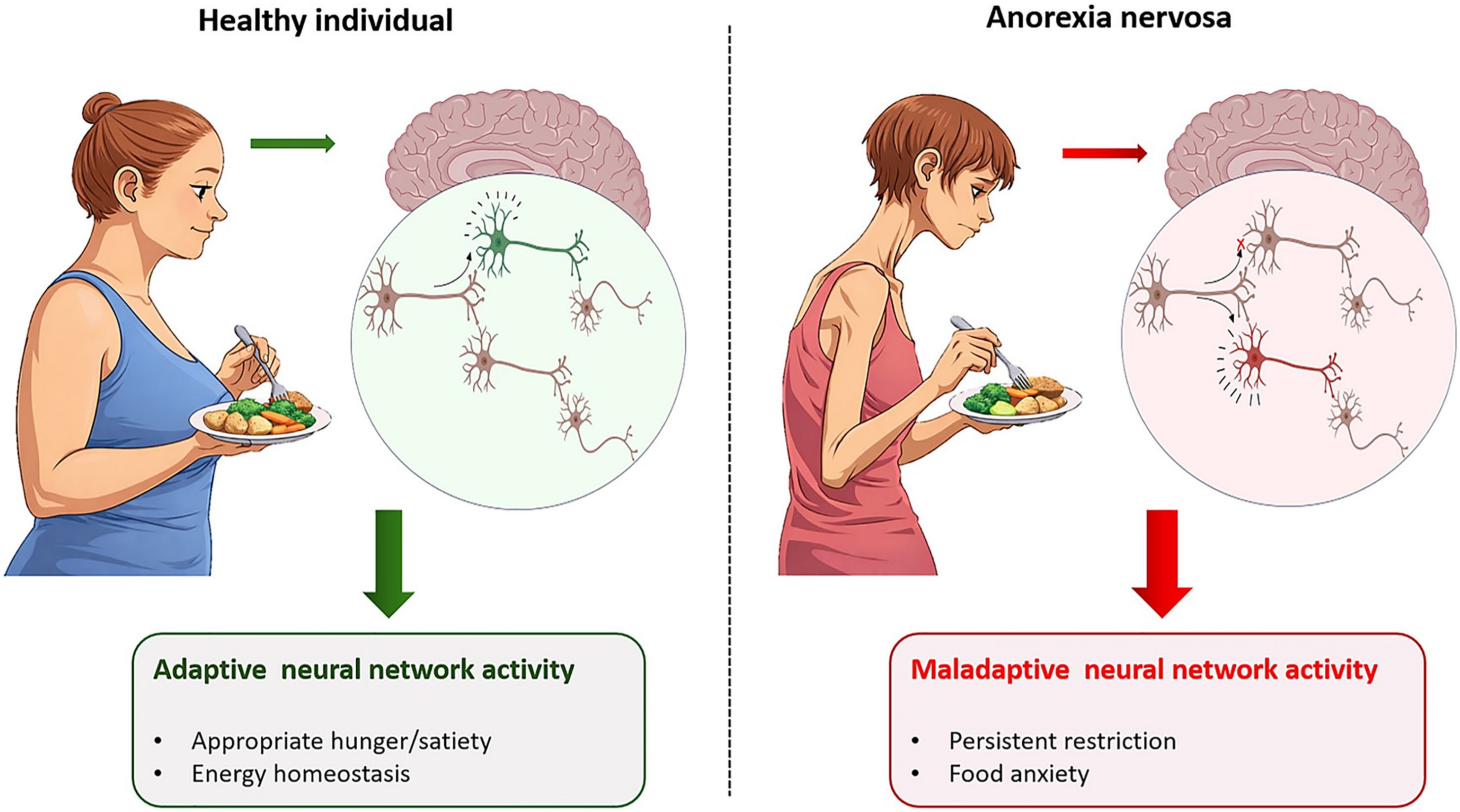
Metabolic modulation of neural network activity in anorexia nervosa. Schematic representation illustrating how identical nutrient intake can engage distinct neural network activity in healthy individuals compared with individuals with anorexia nervosa. In the healthy condition (left), nutrient intake activates an adaptive neural network response in which specific neurons within the network are engaged (green), supporting appropriate hunger–satiety signaling and maintenance of energy homeostasis. In contrast, in anorexia nervosa (right), despite consumption of the same meal, neural activity is recruited in a different subset of neurons (red), reflecting a maladaptive neural network response. This altered neural recruitment is associated with pathological feeding behavior characterized by persistent dietary restriction and increased food-related anxiety. The illustration highlights how metabolic signals can differentially modulate neural network activity, leading to adaptive or maladaptive behavioral outcomes.

While studies on how the brain reacts to actual nutrient intake rather than how it processes visual food cues are rare, they have shown parallel disturbances in circuits that mediate both homeostatic, cognitive, emotional, and reward-based regulation of food intake (Figure 1). Florent et al. (2020) found that patients with AN show paradoxically reduced hypothalamic glutamate/glutamine levels following feeding, contrasting sharply with the expected post-prandial increase seen in controls. This blunted metabolic activation suggests that the hypothalamus fails to register nutrient intake normally, weakening the internal cues that would typically promote satiety and relief after eating. Similarly, Simon et al. (2020) investigated glucose-evoked hypothalamic activity and showed that patients display abnormal amplitude of glucose-induced hypothalamic deactivation compared with controls. They also found that the glucose-dependent connection between the hypothalamus and nucleus accumbens (NAc), a known hub for reward-driven behaviors, was perturbed in AN patients. In fact, patients–but not healthy controls–react with a calorie-dependent increase in fear of being fat and exaggerated satiety even when they were blinded to whether they received water only or different amounts of glucose via gastric infusion (Rigaud et al., 2007). A metabolic-challenge study using 2-deoxy-D-glucose (2-DG), which induces cellular glucopenia, further showed that individuals with AN fail to exhibit the robust hunger response normally evoked by 2-DG. In contrast, healthy controls showed marked increases in hunger and physiological arousal (Nakai et al., 1987). Clinical measures of appetite similarly reveal blunted hunger–satiety modulation in AN patients (Klastrup et al., 2020). Furthermore, broad appetite-related brain circuits fail to respond appropriately to metabolic states. Holsen et al. (2012) showed that key reward and interoceptive regions such as the hypothalamus, amygdala, and anterior insula are hypoactive during hunger in AN. In healthy controls, anterior insula activity correlated positively with hedonic food ratings and this relationship was absent in AN, suggesting disrupted integration of neural responses with hedonic valuation of food. This muted activation persists even after weight restoration, suggesting that the metabolic-neural uncoupling is not simply an acute effect of starvation and body weight loss. Indeed, cerebral blood-flow responses to fasting and feeding remain abnormal despite normalized weight (Wierenga et al., 2017). Westwater et al. (2022) showed that individuals with AN exhibit altered levels of glutamate, myo-inositol, and N-acetylaspartate in prefrontal and occipital cortices, metabolites tied to mitochondrial function and axonal glial signaling. These altered profiles correlate with rigid, automatic eating-related habits, suggesting that starvation-induced metabolic remodeling of these circuits impairs flexible, goal-directed control over behavior.

Together, these findings highlight a fundamental disruption of central energy-sensing mechanisms in AN, characterized by persistent failure to translate metabolic need into hunger with positive valence.

### 2.3 Metabolic dysregulation in anorexia nervosa depends on neurocircuit and synaptic remodeling

AN symptoms can be reproduced experimentally at least partly in animal models. These allow mechanistic investigation of how metabolic imbalance interacts with neural circuits to produce core features of the disorder (Spadini et al., 2021). When rodents are given restricted access to food and unlimited access to a running wheel in the activity-based anorexia (ABA) model, they begin to reduce food intake and increase activity despite progressive weight loss. This paradoxical behavioral coupling closely mirrors the self-starvation and hyperactivity found in AN (Miletta et al., 2021).

Under physiological conditions, agouti-related peptide (AgRP) neurons in the arcuate nucleus of the hypothalamus respond robustly to energy deficit, increasing activity to promote feeding. In the ABA model, however, this adaptive response becomes dysregulated. AgRP neurons exhibit altered excitatory input and abnormal integration of food-related signals despite profound weight loss (Spadini et al., 2021; Miletta et al., 2021). Notably, chemogenetic or optogenetic activation of AgRP neurons increases food intake and attenuates pathological weight loss, whereas disruption of AgRP function exacerbates maladaptive hyperactivity and increases mortality under restricted feeding conditions (Spadini et al., 2021; Miletta et al., 2020; Sutton Hickey et al., 2023). These findings suggest that sustained ABA can destabilize hypothalamic hunger-encoding circuits, leading to a functional uncoupling between physiological energy need and feeding behavior associated with remodeling of synaptic inputs within key hypothalamic metabolic nuclei.

ABA affects not only hypothalamic circuits but also regions governing cognitive control, stress responsivity, and contextual learning. In adolescent animals, ABA disrupts normal maturation of hippocampal CA1 pyramidal neurons. Early increases in dendritic branching are followed by excessive pruning during relapse, suggesting that ABA disrupts normal synaptic maturation (Chowdhury et al., 2014). At the molecular level, ABA increases surface expression of α4βδ-containing GABA_A_ receptors on CA1 dendritic spines, enhancing tonic inhibition and shifting local excitation–inhibition balance (Aoki et al., 2014). Such changes may contribute to increased anxiety-like behavior and reduced behavioral flexibility.

Parallel alterations occur in the medial prefrontal cortex, where ABA reduces dendritic spine density and modifies glutamatergic synaptic composition (Mottarlini et al., 2022). These structural and synaptic changes are associated with impairments in recognition memory and cognitive control. Importantly, some of these alterations persist beyond acute weight loss, suggesting that metabolic stress may induce circuit adaptations with long-term stability. Interventions targeting GABAergic microcircuits have been shown to mitigate pathological hyperactivity and partially normalize feeding in vulnerable animals, further supporting an excitation–inhibition imbalance in the ABA phenotype (Aoki and Santiago, 2022).

Reward-related circuits are likewise sensitive to metabolic challenge. Distinct patterns of plasticity-associated molecules, including brain-derived neurotrophic factor (BDNF) and neural cell adhesion molecule 1, are observed in the ventral tegmental area (VTA), hippocampus, and prefrontal cortex following scheduled feeding and exercise characteristic of ABA (Ho et al., 2016). These coordinated molecular changes suggest that negative energy balance alters these mesocorticolimbic circuits involved in motivation for food and physical activity.

A case report and a translational review have furthermore suggested that ketogenic interventions (alone or combined with ketamine) may influence core AN symptoms (Scolnick et al., 2020) and underlying brain mechanisms (Frank and Scolnick, 2024), though evidence remains preliminary. Consistent with this, ketogenic food has been reported to blunt key ABA behaviors (Dong et al., 2025a), and relapse-focused ABA work has further linked ketogenic (± ketamine) protection to hippocampal synaptic ultrastructure (Dong et al., 2025b).

Collectively, evidence from the ABA model indicates that ABA is sufficient to induce synaptic remodeling across hypothalamic, cortical, and limbic circuits. Although extrapolation to human AN requires caution, these findings suggest that AN depends on structural and functional adaptations within neural circuits that regulate hunger, anxiety, cognitive control, and reward that may outlast acute changes in the metabolic state.

## 3 Neural circuits integrate metabolism with emotional and cognitive aspects of behavior

Anatomical mapping and functional interrogation of genetically defined neurocircuitry show dense bidirectional connectivity between metabolic hubs in the hypothalamus and brainstem and limbic and cortical brain regions. This indicates that brain–body control of metabolic state is not regulated in isolation but is continuously integrated with emotional, reward and cognitive processes (Rossi and Stuber, 2018).

Work dissecting the control of feeding has shown that classic homeostatic circuits in the hypothalamus, which encode energy deficit and nutrient status, overlap with reward-processing regions such as the VTA and NAc. This allows metabolic signals to influence motivation and affective responses to food (Rossi and Stuber, 2018; Bond et al., 2020). Elegant circuit-level studies further demonstrate that midbrain dopaminergic pathways encode palatability and hedonic drive while being modulated in ways that couple energy balance to emotional and motivational states (Zhu et al., 2025).

In reverse, hedonic eating, driven by palatability rather than caloric need, has been linked to a peri–locus coeruleus–to-VTA circuit in which VTA dopamine neurons encode palatability and bidirectionally regulate intake of rewarding foods. This circuit connects arousal and reward systems to metabolic state (Zhu et al., 2025). Other projections to the VTA from the NAc medial shell are inhibited during food seeking and consumption. Experimental manipulation of this pathway can both suppress and potentiate feeding. These data suggest that the same neural circuitry that mediates emotional reward valuation also governs the motivational expression of metabolic need (Bond et al., 2020).

In the arcuate nucleus of the hypothalamus, AgRP neurons offer another neuronal link between metabolic state and affective–motivational drive. These neurons are sufficient to rapidly affect feeding behavior (Aponte et al., 2011) and receive widespread input from brain regions involved in energy homeostasis (Aponte et al., 2011; Wang et al., 2015). However, whole-brain mapping shows that AgRP neurons receive direct input also from limbic and cortical regions involved in stress, learning and emotion, explaining how hunger and satiety can be modulated by anxiety, attention and decision-making in humans (Rossi and Stuber, 2018; Wang et al., 2015). Indeed, optogenetic activation of a small AgRP population in satiated mice triggers immediate, intense feeding, illustrating that hunger signals are encoded as powerful motivational states rather than minor homeostatic adjustments (Aponte et al., 2011).

Hence, the brain integrates metabolic information in the same distributed circuits that regulate mood, motivation and cognition, rendering energy balance inseparable from the emotional and cognitive aspects of behavior it helps shape.

## 4 Metabolic signals in AN affect food intake and synaptic plasticity

What and how different brain areas communicate depends on synaptic plasticity, the activity-dependent modification of synapse number and strength. Plasticity is coordinated by structural and molecular modifications of dendritic spines and presynaptic terminals that regulate synapse efficacy and connectivity (Holtmaat and Svoboda, 2009). These plastic changes are energetically expensive: they require sustained ion pumping, protein synthesis, cytoskeletal remodeling and membrane trafficking (Karbowski, 2019). In fact, synaptic plasticity consumes a non-trivial fraction of the brain’s energy budget, implying that the nervous system must balance changes in how different brain areas communicate against available metabolic resources (Karbowski, 2019). In addition to absolute energetic demands, synaptic plasticity is dynamically regulated by systemic metabolic signals that convey information about nutritional and hormonal state. Work on hippocampal, hypothalamic and brainstem circuits have demonstrated that synaptic organization within these networks is itself plastic and modulated by nutritional and hormonal status. AN is accompanied by widespread endocrine and metabolic disturbances that reflect chronic energy deficiency and affect virtually every part of the hypothalamic–pituitary axis (Støving, 2019; Schorr and Miller, 2017). Hypogonadotropic hypogonadism with functional hypothalamic amenorrhea is typical, driven by reduced GnRH and gonadotropin secretion and resulting in hypoestrogenism (Støving, 2019; Schorr and Miller, 2017). Patients also show a characteristic “starvation profile” with hypoglycemia, hypercortisolemia and growth hormone resistance (elevated GH but low IGF-1) (Støving, 2019; Schorr and Miller, 2017). Adipokine and gut-hormone profiles are also markedly altered, with low leptin and insulin but elevated ghrelin and peptide YY. In metabolically healthy individuals these patterns promote hyperphagia in starvation; paradoxically, in AN, they persist alongside sustained dietary restriction, reflecting a dissociation between peripheral metabolic signaling and behavior (Støving, 2019; Schorr and Miller, 2017). Although many of these abnormalities improve with weight restoration, some can persist for years (Støving, 2019; Schorr and Miller, 2017).

Together, these findings suggest that the metabolic environment characteristic of AN may alter both the energetic constraints on synaptic plasticity and the endocrine signaling cues that regulate synaptic remodeling, with downstream consequences for the organization and persistence of feeding-related circuit function (Spadini et al., 2021; Holtmaat and Svoboda, 2009; Karbowski, 2019; Zeltser et al., 2012).

### 4.1 Glucose metabolism—both glucose and insulin matter

Glucose is the primary energetic substrate for neuronal activity. Its regulated delivery is essential for sustaining synaptic transmission and activity-dependent plasticity (Mergenthaler et al., 2013). It is also a metabolic signal that influences neuronal function apart from supplying energy. Central glucose-sensing networks in the hypothalamus have long been known to monitor fluctuations in peripheral nutrient availability via intracellular signaling cascades that regulate neuronal excitability (Ferrario and Reagan, 2018). Emerging evidence indicates that glucose links nutrient availability with satiety at least in part by regulating excitatory synaptic plasticity within feeding circuits, as discussed further below in the section on the posttranslational modification O-GlcNAc.

The role of glucose is modulated by insulin not only through insulin’s regulation of systemic glucose levels but also its direct effect on food intake and synaptic plasticity. Targeted deletion of neuronal insulin receptors leads to hyperphagia, impaired fuel handling, and altered body-weight regulation, demonstrating that intact central insulin signaling is necessary for maintaining energy homeostasis (Brüning et al., 2000).

In hippocampal circuits, insulin affects PI3Kinase-dependent pathways that adjust the threshold for long-term potentiation (LTP) and depression (LTD). This tunes the synaptic plasticity rules that support memory formation (van der Heide et al., 2005). Electrophysiological studies further demonstrate that physiological insulin concentrations can shift AMPA- and NMDA receptor-mediated signaling in a glucose-dependent manner, facilitating LTP when substrate availability is adequate (Zhao et al., 2019). These findings indicate that insulin not only reflects metabolic status but also directly shapes activity-dependent synaptic remodeling (Ferrario and Reagan, 2018).

Additionally, in humans, intranasal insulin reduces hedonic valuation of food cues and dampens mesolimbic reward responses to palatable foods (Tiedemann et al., 2017). Complementary rodent experiments demonstrate that NAc insulin signaling suppresses food intake and decreases effort-based motivation for palatable foods (Finnell and Ferrario, 2022). Within hypothalamic AgRP circuits, enhanced insulin signaling preferentially reduces consumption of energy-dense foods and mitigates diet-induced insulin resistance (Dodd et al., 2021).

Together, these data show that glucose and insulin integrate metabolic status with synaptic and circuit-level processes governing food valuation, reward signaling, and energy balance (Ferrario and Reagan, 2018).

### 4.2 Ketones: an energy deprivation signal affecting synaptic function

During fasting, caloric restriction and ketogenic states, circulating levels of the ketone body *β*-hydroxybutyrate (BHB) rise substantially. Elevated circulating BHB has also been documented in individuals with AN (Peters et al., 1998; Pahl et al., 1985). While traditionally recognized as an alternative fuel to glucose, BHB is also a signaling molecule capable of reshaping transcription, synaptic transmission and plasticity in brain networks that influence ingestive behavior (Shimazu et al., 2013; Sleiman et al., 2016; Izumi et al., 1998; Bredvik et al., 2024). For example, Shimazu et al. (2013) demonstrated that BHB is an endogenous and relatively specific inhibitor of class I histone deacetylases, such that exogenous BHB administration, fasting and calorie restriction all increase global histone acetylation in mouse tissues and drive transcriptional programs that enhance oxidative stress resistance, including upregulation of Foxo3a and metallothionein-2. By linking a metabolite generated in states of negative energy balance to epigenetic regulation of gene expression, this work shows how metabolic status can durably alter neuronal function and stress resilience in ways that are likely to influence the long-term stability of synapses within feeding-related circuits (Shimazu et al., 2013). Sleiman et al. (2016) extended this concept by showing that prolonged voluntary exercise leads to accumulation of BHB in the hippocampus. In the hippocampus BHB functions as an endogenous inhibitor of histone deacetylases HDAC2 and HDAC3 and increases BDNF expression. Given that BDNF is a major facilitator of glutamatergic synaptic plasticity, including LTP, these findings suggest a pathway through which systemic metabolic signals arising from altered substrate utilization can modulate synaptic strength and plasticity (Sleiman et al., 2016).

### 4.3 Combined effects of body-to-brain hormonal signaling

Diet- and starvation-related shifts in energy balance trigger coordinated adjustments across multiple hormonal systems. It is the combined action of these signals—rather than any single factor—that shapes feeding behavior and the synaptic plasticity of feeding circuits.

Ghrelin exemplifies how nutrient-sensitive hormones influence both homeostatic and higher cognitive circuits. Its central administration rapidly increases feeding and activates homeostatic arcuate neurons (Nakazato et al., 2001). However, its effects on motivated responding for palatable food depend on dopaminergic signaling between the VTA and NAc. Thus, ghrelin’s target circuit dissociates its modulation of reward valuation from its impact on caloric intake (Skibicka et al., 2013). Ghrelin also directly modulates plasticity: it increases dendritic spine density, enhances LTP and improves spatial learning, whereas ghrelin deficiency produces impaired CA1 structural plasticity and memory deficits that are reversed by hormone replacement (Diano et al., 2006). These findings show that fasting-related rises in ghrelin, accompanied by falling leptin and other satiety signals, jointly regulate homeostatic feeding, reward-based decisions and learning about food-related environments (Nakazato et al., 2001; Skibicka et al., 2013; Diano et al., 2006; Sumithran et al., 2011; Hebebrand et al., 2022).

Sex- and adiposity-dependent hormones further shape synaptic plasticity within feeding and cognitive circuits. Estradiol fluctuations enhance hippocampal LTP and reduce LTD during proestrus (Good et al., 1999; Kramár et al., 2013). Estradiol also rewires hypothalamic melanocortin circuits, increasing excitatory synapses onto pro-opiomelanocortin neurons and reducing food intake even in the absence of intact leptin signaling. This indicates that reproductive state modulates energy balance through synaptic mechanisms overlapping with leptin pathways (Kramár et al., 2013; Gao et al., 2007). Furthermore, central adiponectin reduces body weight by increasing energy expenditure, complementing leptin and ghrelin actions yet acting through distinct receptor systems that converge on weight regulation (Qi et al., 2004).

In humans, weight-loss studies demonstrate that reductions in leptin, peptide YY, cholecystokinin and insulin, combined with elevated ghrelin, persist long after initial weight loss, maintaining a hormonal environment that promotes increased hunger and efficient energy storage (Sumithran et al., 2011). This same endocrine pattern engages starvation-related behavioral and cognitive responses, reinforcing the view that synaptic plasticity within hypothalamic, mesolimbic and hippocampal circuits is shaped by the ensemble of hormonal signals generated by nutrient status, adiposity and reproductive physiology (Skibicka et al., 2013; Diano et al., 2006; Hebebrand et al., 2022; Gao et al., 2007). Across these studies, hormones regulating appetite, metabolism and sex state act in concert to influence both food intake and the synaptic architecture that encodes learning and motivation, showing that metabolic and hormonal integration is fundamental to the neural plasticity underlying feeding behavior (Nakazato et al., 2001; Sumithran et al., 2011; Hebebrand et al., 2022; Qi et al., 2004). A summary of key systemic metabolic signals and their synaptic effects in AN is presented in Table 1.

**Table 1:** Key systemic metabolic signals and their synaptic effects in AN.

Metabolic signal	Direct effects	Indirect effects
Glucose	Regulates neuronal excitability; linked to satiety partly by regulating excitatory synaptic plasticity in feeding circuits.	Sets energetic constraints on plasticity (as primary neuronal substrate), shaping capacity for synaptic remodeling.
Insulin	PI3K-dependent tuning of LTP/LTD thresholds; shifts AMPA/NMDA signaling to facilitate LTP when substrate is adequate.	Integrates metabolic status with circuit-level food valuation/reward and energy balance.
*β*-hydroxybutyrate	HDAC inhibition → ↑ histone acetylation → transcription for oxidative stress resilience; in hippocampus HDAC2/3 inhibition → ↑ BDNF → supports glutamatergic plasticity (incl. LTP)	Durable “negative energy balance” signal that can stabilize long-term synaptic/circuit adaptations via gene-expression programs
Ghrelin	↑ dendritic spine density, ↑ LTP, improves learning; deficiency impairs CA1 structural plasticity, reversible with replacement	Acts through dopaminergic VTA–NAc signaling to modulate motivated responding for palatable food (reward-circuit modulation)
Leptin		Part of the combined starvation endocrine pattern (low leptin with elevated ghrelin etc.) proposed to shape feeding-circuit function/plasticity as an ensemble
Peptide YY		Included in the endocrine ensemble altered in AN and after weight loss; implicated as part of the persistent hormonal milieu influencing feeding-related circuits
Estradiol	↑ hippocampal LTP, ↓ LTD; rewires hypothalamic melanocortin circuits by ↑ excitatory synapses onto POMC neurons	Overlaps with leptin-related regulation of energy balance via synaptic mechanisms (reproductive state modulating feeding circuitry)
Adiponectin		Central adiponectin reduces body weight by ↑ energy expenditure; complementary hormonal signal converging on weight regulation
Cortisol		Part of the starvation endocrine profile (hypercortisolemia) contributing to the metabolic milieu proposed to alter constraints/cues for plasticity
Growth hormone/IGF-1 axis		Starvation profile includes GH resistance (↑GH, ↓IGF-1), contributing to the endocrine milieu proposed to alter constraints/cues for plasticity

PI3K, phosphoinositide 3-kinase; LTP, long-term potentiation; LTD, long-term depression; AMPA. *α*-amino-3-hydroxy-5-methyl-4-isoxazolepropionic acid; NMDA, N-methyl-D-aspartate; HDAC, histone deacetylase; BDNF, brain-derived neurotrophic factors; CA1, cornu ammonis field 1 of the Hippocampus; VTA, ventral tegmental area; NAc, nucleus accumbens; POMC, proopiomelanocortin; GH, growth hormone; IGF-1, insulin-like growth factor 1.

## 5 Metabolic sensors translate metabolic signals to altered synaptic plasticity

**Figure 2:**
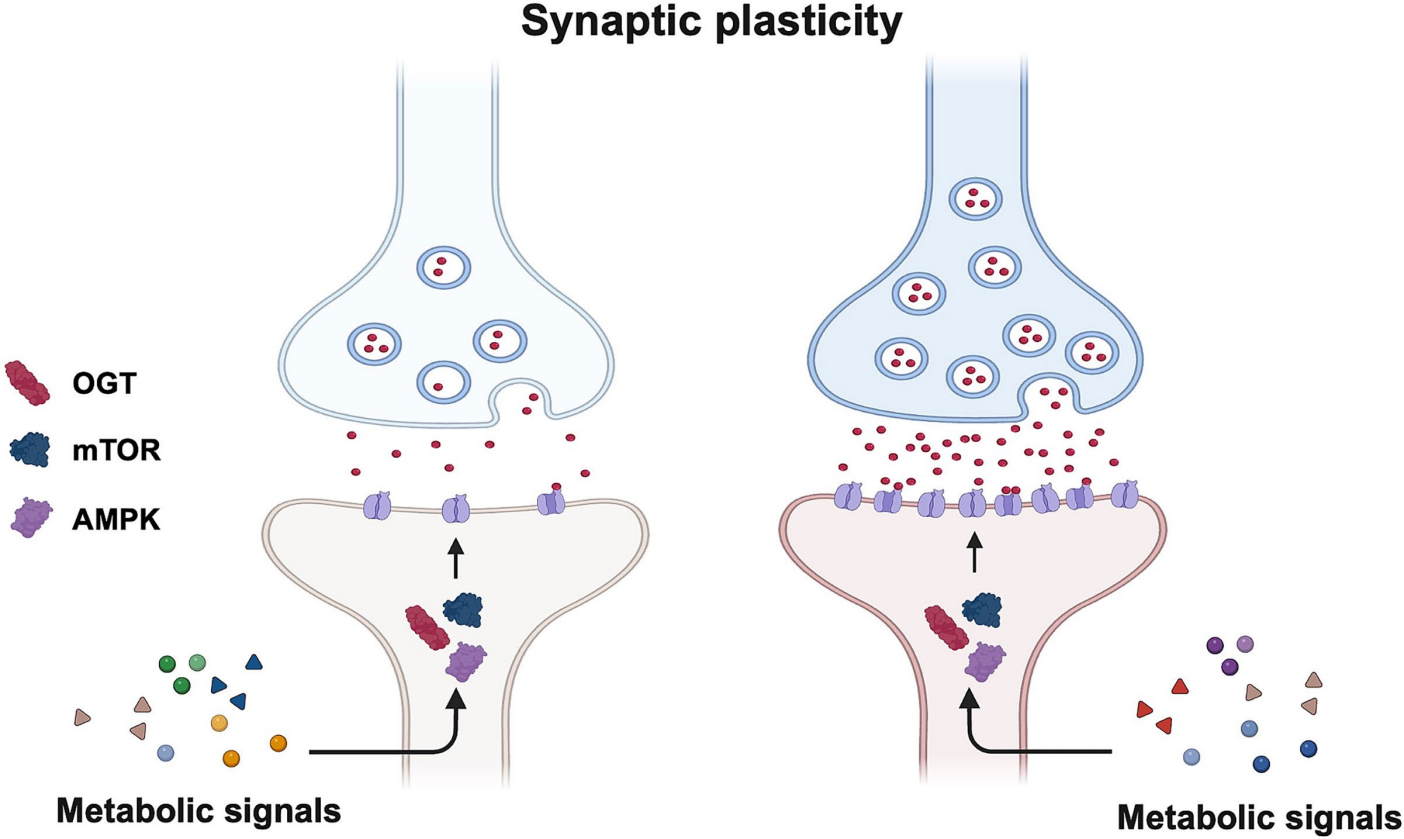
Metabolic regulation of synaptic plasticity. Synaptic plasticity is dynamically regulated by systemic metabolic signals, both nutritional and hormonal (i.e., glucose, insulin, leptin, and ghrelin). These affect synaptic plasticity through intracellular metabolic sensors, such as AMP-activated protein kinase (AMPK), mechanistic target of rapamycin (mTOR), and O-GlcNAc transferase (OGT). Thus, mTOR, AMPK, and OGT translate oscillations in metabolic flux to changes in neuronal communication.

Neurons express several metabolic sensors that detect and translate metabolic signals to altered circuit function by regulating synaptic plasticity. Thus, these sensors control what neurons communicate with each other in response to changes in metabolic flux (Figure 2).

### 5.1 AMPK

AMP-activated protein kinase (AMPK) is a highly conserved serine/threonine kinase that gauges metabolic flux by sensing changes in the AMP/ATP and ADP/ATP ratios. It is abundantly expressed in neurons where it links energy status to synaptic function and behavior (Nicol and Ma, 2025). When oxidative metabolism cannot keep up with activity-dependent ATP demand—for example during synaptic firing, fasting, or hypoglycemia—rising AMP and ADP activate AMPK through allosteric binding and phosphorylation. Increased AMPK activation then affects ion pumping, protein synthesis, autophagy, and mitochondrial function (Nicol and Ma, 2025; Marinangeli et al., 2018). In the hypothalamus, AMPK integrates intracellular energy status with circulating hormones and nutrients in key appetite-controlling nuclei. Orexigenic signals like ghrelin and hypoglycemia activate hypothalamic AMPK, whereas anorexigenic signals such as leptin, insulin, and refeeding inhibit AMPK (Nicol and Ma, 2025; Shimizu et al., 2008). Rodent studies have shown that bidirectional manipulation of hypothalamic AMPK is sufficient to alter food intake and body weight (Shimizu et al., 2008). Work in AgRP neurons have revealed that fasting-induced activation of AMPK drives a postsynaptic AMPK → p21-activated kinase (PAK) signaling cascade that is required for dendritic spinogenesis and increased excitatory synaptic input onto these neurons. This encodes prolonged negative energy balance as a structural strengthening of hunger circuits rather than as a transient change in firing rate alone (Kong et al., 2016). Inhibiting AMPK or PAK in AgRP neurons prevents fasting-induced spine formation and blunts the associated increase in excitatory drive and feeding, whereas activating this pathway in otherwise fed animals can mimic the synaptic and behavioral effects of food deprivation, indicating that AMPK-sensitive remodeling of synaptic connectivity is a key mechanism by which metabolic flux shapes orexigenic drive (Kong et al., 2016).

A hypothalamic “flip–flop” memory network that stabilizes hunger and satiety states has been shown to rely on AMPK-dependent synaptic plasticity to encode transitions in energy status over time (Yang et al., 2011). In this model, changes in nutrient and hormonal availability modulate AMPK activity in interconnected excitatory and inhibitory neurons, which in turn modifies synaptic strength in a positive feedback loop that locks the system into either a hunger or satiety state that persists beyond immediate metabolic signals (Yang et al., 2011).

In hippocampal and cortical networks, AMPK also couples local metabolic flux to synaptic plasticity and memory by regulating ATP supply during activity and controlling catabolic and anabolic processes at synapses (Nicol and Ma, 2025; Marinangeli et al., 2018). Marinangeli et al. (2018) showed that synaptic activation increases neuronal oxygen consumption and oxidative phosphorylation in an AMPK-dependent manner. This metabolic response is necessary for the induction of immediate-early gene expression, LTP, and normal performance in learning tasks. However, context and intensity of activation are critical: Domise et al. (2019) demonstrated that chronic or excessive AMPK activation in primary neurons induces autophagy-mediated degradation of pre- and postsynaptic proteins, leading to loss of synapses and reduced network activity.

In sum, by detecting changes in metabolic flux and orchestrating adaptive or maladaptive responses in synaptic structure and function, AMPK serves as a metabo-synaptic integrator that links energy balance to both food intake and the capacity of neural circuits to undergo plasticity (Nicol and Ma, 2025; Marinangeli et al., 2018; Shimizu et al., 2008; Kong et al., 2016; Yang et al., 2011; Domise et al., 2019).

### 5.2 mTOR

The mechanistic target of rapamycin (mTOR) is a serine/threonine kinase that detects metabolic flux in the brain by integrating signals from ATP levels, amino acids, glucose, oxygen and growth factors into changes in protein synthesis, autophagy and cell growth. It couples cellular energy status to neuronal excitability and synaptic function (Bockaert and Marin, 2015; Blouet et al., 2008). The mTOR functions in two main complexes, mTORC1 and mTORC2. The mTORC1 is most closely linked to nutrient sensing and translational control at synapses, where it regulates cap-dependent translation initiation through downstream effectors such as 4E-binding proteins and S6 kinases to adjust the local availability of plasticity-related proteins in an activity- and metabolism-dependent fashion (Bockaert and Marin, 2015; Tang et al., 2002; Morrison et al., 2007). In long-lasting forms of synaptic plasticity, including late-phase LTP and certain forms of LTD, inhibition of the mTOR pathway prevents the protein-synthesis-dependent maintenance of synaptic change. mTOR activity is required to stabilize potentiated or depressed states beyond the initial induction period and thus for consolidating memory traces in hippocampal and cortical circuits (Blouet et al., 2008; Tang et al., 2002; Hoeffer et al., 2008). Subsequent work has shown that synaptic mTOR signaling is finely tuned rather than simply “on” or “off,” with both insufficient and excessive activity impairing plasticity (Bockaert and Marin, 2015; Mori et al., 2009; McCabe et al., 2020).

Developmental and stress models further link mTOR to structural synaptic remodeling. In rodents, environmental insults that chronically reduce or dysregulate mTOR signaling in hippocampus and prefrontal cortex are associated with decreased levels of postsynaptic density proteins, dendritic spine loss and impaired LTP. In reverse, targeted restoration of mTOR activity rescues synaptic protein synthesis, plasticity and cognitive performance. This shows that mTOR integrates the effect of metabolic, stress and neuromodulatory pathways on circuit function (Hoeffer et al., 2008; Wang et al., 2020; Dagon et al., 2012; Martins et al., 2012).

mTOR signaling in hypothalamic neurons regulates food intake and body weight (Cota et al., 2006; Dwyer and Duman, 2013). In the hypothalamus, mTORC1 activity increases in response to amino acids such as leucine and to adiposity signals like leptin. Experimental activation of mTOR in the mediobasal hypothalamus decreases food intake and body weight. Pharmacological inhibition blunts the anorectic effects of leptin and promotes feeding (Cota et al., 2006; Dwyer and Duman, 2013; Haissaguerre et al., 2014). Genetic and pharmacological studies further suggest that distinct hypothalamic cell populations use mTOR to integrate circulating nutrients and hormones with local energy demand. This shapes the excitatory–inhibitory balance in orexigenic and anorexigenic circuits and influences both meal size and long-term energy balance (Dwyer and Duman, 2013; Haissaguerre et al., 2014; Hilal et al., 2025).

Thus, mTOR signaling regulates energy homeostasis and learning processes that govern motivated feeding behavior (Bockaert and Marin, 2015; Mori et al., 2009; Cota et al., 2006; Dwyer and Duman, 2013; Hilal et al., 2025).

### 5.3 O-GlcNAc

O-GlcNAcylation is a nutrient-sensitive posttranslational modification in which a single N-acetylglucosamine moiety is added to serine and threonine residues on cytoplasmic and nuclear proteins via the Hexosamine Biosynthetic Pathway (HBP). HBP flux depends on glucose, amino acids, fatty acids and nucleotide availability, making O-GlcNAc levels depend on cellular metabolic state. In addition to nutrients, several metabolic hormones including ghrelin and insulin can directly affect O-GlcNAc (Lagerlöf, 2018; Uygar and Lagerlöf, 2023). The brain is one of the organs where O-GlcNAc is most abundant. In the brain, O-GlcNAc is dynamically regulated across development, adulthood and aging. Hundreds of synaptic and nuclear proteins are modified with O-GlcNAc. A single enzyme adds O-GlcNAc to proteins–O-GlcNAc transferase (OGT)–and there is only one enzyme that removes it, O-GlcNAcase (OGA). Through these two enzymes, O-GlcNAc cycling on and off proteins integrates multiple metabolic pathways, transcriptional programs and synaptic function (Lagerlöf, 2018; Uygar and Lagerlöf, 2023; Andersson et al., 2021).

It was first demonstrated in the hippocampus that O-GlcNAc is required for LTP (Tallent et al., 2009). More targeted studies indicated that O-GlcNAc cycling affects synaptic plasticity at least in part through GluA2-containing AMPA receptors, linking nutrient-driven O-GlcNAc changes to the core receptor machinery that encodes dynamically regulated synaptic strength (Taylor et al., 2014). Genetic or pharmacological perturbation of O-GlcNAc cycling in the hippocampus–either by reducing OGA activity and thereby globally elevating O-GlcNAc levels, or by diminishing OGT function–impairs both LTP and LTD and leads to deficits in spatial learning and memory. This demonstrates that synaptic plasticity critically depends on the level and timing of O-GlcNAcylation (Tallent et al., 2009; Taylor et al., 2014; Yang et al., 2017). Consistent with the requirement for tightly tuned O-GlcNAc signaling, conditional modulation of neuronal O-GlcNAc in aging mice shows that restoring age-related declines in OGT expression and protein O-GlcNAcylation improves synaptic function and cognitive performance (Lagerlöf, 2018; Uygar and Lagerlöf, 2023; Wheatley et al., 2019).

O-GlcNAc cycling controls not only synaptic strength but also structural maturation of excitatory synapses and dendritic spine morphogenesis. OGT is enriched in the postsynaptic density (PSD) of excitatory synapses, where neuronal stimulation increases O-GlcNAcylation on multiple PSD proteins. Loss of OGT reduces synaptic GluA2/GluA3 content, decreases spine density and results in immature excitatory synapses. Thus, O-GlcNAc is a sensor that couples neuronal activity and metabolic status to synapse development and stability (Lagerlöf et al., 2017). Conversely, OGA activity is required for normal dendritic spine growth and maintenance; altered O-GlcNAc turnover disrupts spine morphology and glutamatergic transmission, further underscoring that balanced cycling—rather than unidirectional modification—is essential for healthy synaptic plasticity (Yang et al., 2017; Han et al., 2026).

These O-GlcNAc-dependent synaptic mechanisms are directly connected to neural control of feeding and energy balance. In the paraventricular nucleus of the hypothalamus (PVN), fasting decreases and high glucose increases O-GlcNAc levels. Feeding and higher nutrient availability affect O-GlcNAc levels and drive a robust excitatory synaptic input onto these satiety-related neurons. Deletion of OGT in PVN neurons abolishes feeding-induced synaptic potentiation and leads to hyperphagia and obesity, demonstrating that O-GlcNAc couples metabolic flux to synaptic plasticity that encodes satiety signals (Lagerlöf et al., 2016; Pérez-Del-Pozo et al., 2026). Whereas the effect of long-term deletion of OGT in these neurons may be compensated by the brain through alternative circuitry, manipulations of OGA have, similarly, been shown to affect satiety (Andersson et al., 2021; Dai et al., 2018). In contrast to the downregulation of O-GlcNAc levels in the PVN upon fasting, a study in AgRP neurons observed that OGT and O-GlcNAc in these cells are upregulated by fasting. Loss of OGT reduces AgRP neuron excitability, promotes browning of white adipose tissue and protects against diet-induced obesity (Ruan et al., 2014). While the effect on whole-body metabolism is unclear, manipulations of O-GlcNAc cycling in the VTA affected the animal’s operant response to glucose (Shao et al., 2022; Lee et al., 2020). Thus, O-GlcNAc cycling regulates energy homeostasis by different mechanisms depending on what circuit it affects. Taken together, O-GlcNAc cycling regulates synaptic plasticity and operates across multiple circuits to align energy homeostasis, reward and cognitive processing with the organism’s metabolic state (Lagerlöf, 2018; Uygar and Lagerlöf, 2023; Tallent et al., 2009; Taylor et al., 2014; Yang et al., 2017; Wheatley et al., 2019; Lagerlöf et al., 2017; Han et al., 2026; Lagerlöf et al., 2016; Pérez-Del-Pozo et al., 2026; Ruan et al., 2014).

## 6 Discussion/Conclusions

Recent advances in AN-research reconceptualize AN as a metabo-psychiatric disorder in which metabolic biology is intrinsically linked to psychiatric vulnerability. While historically attributed primarily to psychological and social factors–an interpretation likely influenced, and perhaps erroneously so, by the higher prevalence in females–accumulating evidence from genetics, neuroimaging, physiology, and animal models suggest that metabolic factors in AN shape the communication between brain areas, resulting in aberrant cognition, affect, and behavior.

Across brain regions, metabolic hormones such as insulin, ghrelin, leptin and adiponectin together with nutrient availability affect a set of intracellular sensors—including AMPK, mTOR and the O-GlcNAc system—that translate metabolic fluctuations into synaptic remodeling by regulating protein synthesis, receptor trafficking and structural plasticity. This effect on synaptic function links starvation-associated endocrine states to long-term changes of circuits governing homeostatic, motivational and cognitive aspects of food intake.

The described signals vary substantially across individuals and by sex, and a key translational implication is that AN is biologically heterogeneous. The clinical presentation varies across subtypes (e.g., restricting vs. binge–purge presentations; presence or absence of compulsive exercise), illness stage (acute underweight, weight restoration, and relapse), and individual profiles (including genetic liability, premorbid metabolic traits, comorbid anxiety and compulsivity, and developmental timing). Within the suggested framework, these dimensions can be conceptualized as partially distinct routes by which metabolic signals and their intracellular sensors remodel synaptic ensembles in different circuits. For example, acute starvation may produce strong state-dependent shifts in excitability and plasticity thresholds, whereas weight restoration may normalize some peripheral signals while leaving more persistent synaptic adaptations in circuits governing interoception, habit learning, and threat processing—thereby contributing to relapse vulnerability. Similarly, symptom dimensions such as hyperactivity/compulsive exercise may reflect stronger engagement of stress- and arousal-linked circuits, while binge–purge symptoms may reflect differential involvement of reward valuation and inhibitory control circuits; in both cases, metabolic signaling could bias the stability and expression of these circuit states via synaptic remodeling mechanisms.

Future work would benefit from defining how metabolic signals reshape specific synaptic ensembles across development, identify how metabolic pathways are modulated by non-metabolic input such as sociocultural pressure to be thin, and determine how targeting metabolic sensors or restoring metabolic–neural coupling that supports synaptic plasticity can yield durable therapeutic benefit. Critically, this agenda will require stratified designs—by sex hormone status, illness stage, and symptom dimensions—combined with multimodal metabolic and circuit readouts to map which metabolic–synaptic mechanisms dominate in which patient subgroups. Because current animal models largely index state-dependent phenotypes without explicitly modeling AN’s polygenic liability, integrating human genetics with these multimodal readouts will be essential to map how inherited risk converges on shared metabolic–synaptic mechanisms. Taken together, this integrated perspective suggests a scientific and clinical trajectory in which treating AN will require interventions that act jointly on metabolic physiology and brain-based processes of learning, motivation, and affect.
